# A plasma metabolomic fingerprint of moderate or severe hearing loss

**DOI:** 10.1007/s11306-026-02395-8

**Published:** 2026-02-27

**Authors:** Yukun Li, Raji Balasubramanian, D. Bradley Welling, Konstantina M. Stankovic, Oana A. Zeleznik, Gary Curhan, Sharon Curhan

**Affiliations:** 1 Department of Biostatistics and Epidemiology, School of Public Health and Health Sciences, University of Massachusetts, Amherst, MA USA; 2 Department of Otolaryngology-Head and Neck Surgery, Massachusetts Eye and Ear, Boston, MA USA; 3 Harvard Medical School, Boston, MA USA; 4 Department of Otolaryngology-Head and Neck Surgery, Stanford University School of Medicine, Stanford, CA USA; 5 Channing Division of Network Medicine, Brigham and Women’s Hospital and Harvard Medical School, Boston, MA USA

**Keywords:** Women, Cohort study, Metabolomics

## Abstract

**Background:**

Disabling hearing loss affects millions of adults world-wide. Metabolomics investigations are comprehensive assessments of an individual’s metabolic processes that could provide insight into biological pathways underlying auditory dysfunction, yet data are limited.

**Methods:**

We conducted a cross-sectional investigation of the association of plasma metabolite profiles and self-reported adult-onset moderate or severe hearing loss among 3925 women, including 1167 hearing loss cases and 2758 controls in the Nurses’ Health Study. Information on hearing status at the time of the blood collection and on relevant risk factors was collected on biennial questionnaires. Metabolic profiling was conducted by liquid chromatography-mass spectrometry. The independent associations of 278 metabolites with hearing loss was assessed in logistic regression models adjusted for age, fasting status, race/ethnicity, co-morbidities, medication use and biobehavioral factors. The false discovery rate was controlled at 5% through the q-value approach. Metabolite Set Enrichment Analysis was conducted to identify metabolite classes that are enriched for concordant associations with hearing loss.

**Results:**

We identified 10 metabolites that were significantly associated (q value < 0.05) with moderate or severe hearing loss in multivariable-adjusted models. Steroid esters were enriched for negative associations, while triglycerides were enriched for positive associations. Triglycerides with fewer double bonds were enriched for significant, positive associations with hearing loss (*p* = 0.04).

**Conclusion:**

In this population-based investigation, we identified that triglycerides were enriched for positive associations, while steroid esters were inversely associated with adult-onset moderate or severe hearing loss. This study indicates that metabolic perturbations may contribute to the pathoetiology underlying adult-onset hearing loss.

**Supplementary Information:**

The online version contains supplementary material available at 10.1007/s11306-026-02395-8.

## Introduction

Hearing loss is highly prevalent worldwide. In 2015, approximately 500 million people worldwide had disabling hearing loss, representing 6–8% of the global population (Wilson et al. 2017). The adverse impact of hearing loss on communication, health and quality of life is considerable and the economic costs are substantial (Dalton et al. 2003; McDaid et al. 2021). Identifying potentially modifiable factors that contribute to hearing loss could inform strategies for prevention and could have direct implications for improving population health (Wilson and Tucci 2021).

Metabolomics investigations are a comprehensive assessment of an individual’s metabolic processes that can provide insight into biological pathways underpinning neurodegenerative conditions, including age-related hearing loss. Identification of plasma metabolomic biomarkers has emerged as an important platform to understand pathophysiologic processes. Metabolite profiling is obtained through analytical techniques including gas chromatography-mass spectrometry (GC-MS) and liquid chromatography-mass spectrometry (LC-MS) (Beale et al. 2018; Theodoridis and Wilson 2008). There is a limited literature on metabolomics of hearing loss. In animal models, alterations in brain metabolite profiles were observed following acoustic trauma (He et al. 2017). A small (*n*= 124) plasma metabolomics investigation of hearing loss in male workers in China exposed to occupational noise found metabolite alterations in men with noise-induced hearing loss (Miao et al. 2021). Other metabolomics investigations in humans have examined urine and perilymph, but sample sizes have typically been small (Malesci et al. 2023; Boullaud et al. 2022).

We conducted a cross-sectional investigation of the association of individual plasma metabolite profiles and self-reported moderate or severe hearing loss among 3925 women who are participants in the Nurses’ Health Study (NHS). We derived a metabolite score using a Lasso model to estimate a single-number summary of a set of metabolites that is associated with moderate or severe hearing loss. Metabolite Set Enrichment Analysis (MSEA) was conducted to examine the relations between metabolite classes and moderate or severe hearing loss.

## Methods

### Study population

Data used in our analysis were collected from the Nurses’ Health Study (NHS). The NHS was established in 1976, when 121,700 female registered nurses, aged 30–55 years, from 11 US states, were enrolled. For this reason, all participants in our study were women. Participants complete detailed questionnaires on extensive demographic, health, diet and lifestyle information every 2 years. The follow-up rate exceeds 90% of the eligible person-time. For this study, we included 3925 women with available metabolomics data previously obtained in 14 sub-studies nested within NHS and who also provided information on their hearing status on the 2012 biennial questionnaire (Table S1). Participants who did not respond to the 2012 questionnaire, did not provide information on their hearing status, or reported mild hearing loss, were excluded. To focus on adult-onset hearing loss, participants who reported hearing loss that began before age 30 were also excluded.

In 1989/1990, NHS participants had their blood drawn in sodium heparin tubes and shipped with an ice pack via overnight courier to the laboratory, where it was processed into plasma, red blood cells and white blood cells. Blood samples were divided into small aliquots and were stored at − 130 ℃ or colder in the vapor phase of liquid nitrogen freezers. In 2000–2002, a second blood sample was collected from a subset of NHS participants through the same protocol (Tworoger et al. 2013). If a participant had more than one set of metabolomics data from different blood draws, we used data from the most recent date of blood draw. The study protocol was approved by the institutional review boards of the Brigham and Women’s Hospital and Harvard T.H. Chan School of Public Health, and those of participating registries as required. The return of the self-administered questionnaire and blood sample was considered to imply consent.

### Metabolite assay

Metabolomics assays were performed within 14 studies nested within the NHS (Table S1). Before combining individual metabolomic data across studies, we applied the probit transformation within each study to account for batch effects. Plasma metabolites were profiled at the Broad Institute of MIT and Harvard (Cambridge, MA) using three complimentary liquid chromatography tandem mass spectrometry (LC-MS/MS) methods (Mascanfroni et al. 2015; O’Sullivan et al. 2017; Paynter et al. 2018; Townsend et al. 2013; Bajad et al. 2006).

Hydrophilic interaction liquid chromatography (HILIC) analyses of water-soluble metabolites in the positive ionization mode were conducted using an LC-MS system comprised of a Shimadzu Nexera X2 U-HPLC (Shimadzu Corp.; Marlborough, MA) coupled to a Q Exactive mass spectrometer (Thermo Fisher Scientific; Waltham, MA). Metabolites of intermediate polarity were also profiled using the same method. Negative ionization mode data were acquired using an ACQUITY UPLC (Waters) coupled to a 5500 QTRAP triple quadrupole mass spectrometer (AB SCIEX) running a modified version of the HILIC method described by (Bajad et al. 2006). Plasma lipids were profiled using a Shimadzu Nexera X2 U-HPLC.

For each method and every 20 samples, pooled plasma reference samples were included. We normalized measurements using the ratio of the value of the sample to the value of the nearest pooled reference multiplied by the median of all reference values for the metabolite. Additionally, 2257 quality control (QC) samples were profiled and randomly distributed among the participants’ samples. After QC analysis, the number of metabolites profiled ranged between studies from 86 in the pancreatic cancer study to 412 in the stroke study (Table S1). Of the 598 unique metabolites available for analysis across studies, 278 metabolites had fewer than 30% of samples missing values and were included in the analysis.

#### Stability of metabolite measures

In plasma samples from the NHS, rigorous pilot testing of the Broad Institute metabolomics platform was performed (Townsend et al. 2013). More than 500 known metabolites were measured, including lipids, amino acids, bile acids, carbohydrates, and others. The observed coefficients of variation (CV%) for blinded duplicates were less than 20% for 79% of the metabolites, suggesting that the assay has good to excellent reproducibility. Samples compared after a 0- or 24-hour processing delay after collection (mimicking our blood collections methods), demonstrated that 82% of the metabolites had Spearman correlation or intra-class correlation (ICC) above 0.75, indicating good to high stability after the processing delay. In addition, 71% of metabolites had a Spearman correlation or ICC greater than 0.40 when measured in samples taken less than years apart, indicating acceptable within-person temporal stability. (Townsend et al. 2013)Moreover, in our recent assessment of within-person stability over 10 years, metabolites had a median ICC of 0.4 (Zeleznik et al. 2022a), demonstrating representation of long-term metabolite levels.

### Assessment of hearing loss

Self-reported moderate or severe hearing loss, the primary outcome, was determined based on the participant’s response to the question on the 2012 long-form questionnaire. On this questionnaire, participants were asked, “Do you have a hearing problem?”. Participants were provided with the response options: “no, mild, moderate, severe.” Participants were also asked, “At what age did you first notice a change in your hearing?” We used this information to identify participants with moderate or worse hearing loss in 2012 whose hearing had begun to noticeably change prior to the time of the blood collection. Consistent with the methods previously used in our study of the association of plasma metabolomic biomarkers with prevalent persistent tinnitus (Zeleznik et al. 2023), for this study, we chose a priori to examine hearing loss that was apparent to the participant prior to the blood collection as the primary outcome.

Questionnaire-based assessment of hearing loss among large populations has been found to be reasonably reliable (Ferrite et al. 2011; Schow et al. 1990; Sindhusake et al. 2001). In a validation study, the sensitivity of a single question to assess hearing loss among women in this age group was 95% for detecting moderate hearing loss, defined as the better ear pure tone average hearing thresholds at 0.5, 1, 2, 4 kHz (BEPTA_0.5, 1, 2, 4 kHz_) > 40 dB HL, and 100% for detecting severe hearing loss (BEPTA_0.5, 1, 2, 4 kHz_>60 dB HL), and the specificity was 65% and 64%, respectively (Sindhusake et al. 2001). Evidence suggests that auditory deterioration may not be fully captured by conventional audiometry (Kujawa and Liberman 2009, 2015), thus in real-world settings, self-reported functional hearing ability may provide an ecologically valid assessment of hearing and identify a larger group of adults with meaningful hearing impairment.

Findings on significant associations of a number of risk factors and the risk of self-reported hearing loss using these methods in the NHS and in similar cohorts have been previously published. (Curhan et al. 2021, 2019, 2014; Lin et al. 2020). For this study, we a priori chose to examine moderate or severe hearing loss as the primary outcome (case) to focus on hearing loss that is likely to be the most clinically meaningful and to minimize misclassification. Controls were defined as those who reported having no hearing problem.

### Covariate assessment

Information on covariates was obtained from sub-study questionnaires completed at the time of blood collection and from biennial questionnaires completed nearest to and before the date of the blood collection. In multivariable-adjusted analyses, we adjusted for sub-study endpoint (see Supplement for details) and factors potentially associated with metabolite profiles and hearing loss, including age (continuous, year), fasting status (yes/no), body mass index (BMI) (continuous, kg/m), race/ethnicity (white/others), diabetes mellitus (yes/no), hypertension (yes/no), menopausal status (pre-menopausal/postmenopausal/indeterminate), use of menopausal hormone therapy (MHT) (yes/no, yes if use oral MHT or other MHT types), smoking (never/previous/current), dietary intake (DASH dietary adherence score) (continuous), alcohol intake (g/day), physical activity (continuous, metabolic equivalents from recreational and leisure-time activities per week), regular (≥ 2 days/week) NSAID use (yes/no)), regular (≥ 2 days/week) acetaminophen use (yes/no), and persistent tinnitus (yes/no).

### Statistical analysis

#### Preprocessing and missing values

Since not all case/control studies included in this analysis measured metabolites using all three LCMS platforms, we excluded any metabolite that was missing in greater than 30% of all samples. In total, 278 metabolites were included. Prior to analysis, missing values were imputed by one half the minimum observed if not all samples in the sub-study were missing the metabolite; otherwise, no imputation was performed. These metabolites were log-transformed and standardized to mean 0 and unit variance prior to the analysis.

#### Statistical models

For each of the 278 metabolites, we excluded participants who were either missing that metabolite measurement and/or any of the covariates adjusted for in the model. The association of each metabolite with moderate or severe hearing loss was assessed in logistic regression models: Model 1 adjusted for age, fasting status, and BMI; Model 2 further adjusted for race/ethnicity, diabetes mellitus, hypertension, menopausal status, menopausal hormone therapy use, smoking, dietary intake (DASH dietary adherence score), alcohol intake, physical activity, NSAID use, acetaminophen use, and persistent tinnitus. The false discovery rate was controlled at 5% using the q-value approach (Storey and Tibshirani 2003). Metabolites that satisfied raw p and q value thresholds of 0.05 in Model 1 were evaluated in Model 2. Statistically significant associations were identified for metabolites with p and q values < 0.05 in Model 2.

Lasso regression analysis was then conducted by simultaneously incorporating the set of significant metabolites that satisfied p and q values < 0.05 in Model 2. We estimated a single-number summary, or “metabolite score,” that is associated with moderate or severe hearing loss.

We conducted Metabolite Set Enrichment Analysis (MSEA) (Subramanian et al. 2005)to identify specific metabolite classes that are enriched for concordant associations with moderate or severe hearing loss (Korotkevich et al. 2021). We examined the relationship between carbon chain length, the number of double bonds, and hearing loss in the subset of 62 triglycerides in linear models.

Sensitivity analyses were conducted to examine the possibility that disease outcomes investigated in the case-control sub-studies, such as cancer, may bias the results. We restricted the sample to participants from the Lifestyle Validation Study (LVS), the Racial Differences Study, and to the participants who served as controls (i.e. did not develop cancer or the other disease outcomes of interest in the case-control studies) from the other case–control sub-studies listed in Table S1 in the Supplement.

Further detail regarding the statistical methods is provided in the Supplement.

## Results

The characteristics of the study participants at the time of their most recent blood draw, according to hearing status, are shown in Table 1. We found no strong pairwise correlations among the covariates in Table 1. Pearson correlations between continuous covariates ranged from − 0.16 to 0.18, correlations between continuous and binary covariates from − 0.15 to 0.43, and phi coefficients between binary covariates from − 0.12 to 0.08. Participants with moderate or severe hearing loss were more likely to be older, post-menopausal, and to have persistent tinnitus. The distribution of moderate or severe hearing loss in each sub-study is shown in Supplemental Table S1.

**Table 1:** Characteristics of nurses’ health study participants at time of blood Collection. The study sample included 3925 participants, including 1167 who reported moderate or severe hearing loss

	No Hearing Loss (*n* = 2758)	Moderate or Severe Hearing Loss (*n* = 1167)	*P*-value*
Age, mean (SD), years	54.7 (7.5)	59.1 (6.5)	< 0.01
Fasting status^1^, %	80.6	80.0	0.66
Body mass index, mean (SD) kg/m^2^	25.4 (4.7)	25.5 (4.6)	0.80
Race and ethnicity, White, %	93.1	95.3	0.01
Diabetes, %	19.0	18.4	0.69
Hypertension, %	46.6	47.3	0.72
Post-menopausal, %	62.6	84.0	< 0.01
MHT use^2^, %	42.1	45.9	0.04
Smoking			
- Never, %	48.6	46.6	0.22
- Past, %	41.8	44.8	
- Current, %	9.5	8.7	
DASH^3^ dietary adherence score, mean (SD)	2.9 (1.4)	3.1 (1.4)	< 0.01
Alcohol intake			
None, %	36.4	38.5	0.37
1–14.9 g/d, %	52.6	52.3	
15 + g/d, %	11.0	9.2	
Physical activity, METs/week^4^	17.3 (24.0)	16.5 (24.6)	0.39
Regular NSAID^5^ use^6^, %	38.4	36.4	0.26
Regular acetaminophen use^6^, %	41.2	41.8	0.74
Persistent tinnitus^7^, %	11.5	30.7	< 0.01

^*^ P-values were calculated using the chi-square test for categorical variables, and t-test for continuous variables

^1^ Fasting status > = 8 h

^2^ *MHT* Menopausal hormone therapy

^3^ *DASH* Dietary approaches to stop hypertension

^4^ *METs* Metabolic equivalents from recreational and leisure-time activities

^5^ *NSAID* Non-steroidal anti-inflammatory drugs

^6^ Regular analgesic use defined as 2 or more days per week

^7^ Tinnitus experienced several days per week or daily

A total of 278 metabolites were individually evaluated in a sample of 1,167 hearing loss cases and 2,758 controls. In Model 1 adjusting for age, fasting status, and BMI, 72 of 278 metabolites were significantly associated with moderate or severe hearing loss, at a q value threshold of 0.05. For each of the selected 72 metabolites from Model 1, we then fit a fully adjusted model (Model 2). We identified 10 metabolites that were significantly associated with moderate or severe hearing loss, with q value < 0.05 (Table 2, Figure S1).

**Table 2:** Associations between plasma metabolites and risk of moderate or severe hearing loss among women in the nurses’ health study

Metabolite	Metabolite Sub-class^1^	OR (95% CI)^2^	*P*-value	Q-value
PE^3^ (P-38:5)/PE(O-38:6)	Glycerophosphoethanolamines	0.75 (0.62, 0.91)	4.0e-3	0.043
N6, N6-dimethyllysine	Amino acids, peptides, and analogues	1.27 (1.07, 1.51)	6.7e-3	0.049
phenylacetylglutamine	Amino acids, peptides, and analogues	1.27 (1.08, 1.48)	3.5e-3	0.043
gabapentin	Amino acids, peptides, and analogues	1.34 (1.11, 1.62)	2.7e-3	0.043
PC^4^(P-38:3)/PC(O-38:4)	Glycerophosphocholines	1.47 (1.12, 1.94)	5.9e-3	0.047
1-methylhistamine	Amines	1.62 (1.16, 2.25)	4.2e-3	0.043
PC(36:4)_B	Glycerophosphocholines	1.75 (1.27, 2.42)	6.4e-4	0.027
homoarginine	Amino acids, peptides, and analogues	1.82 (1.22, 2.73)	3.5e-3	0.043
ribothymidine	Pyrimidine nucleosides	1.98 (1.33, 2.95)	7.6e-4	0.027
1-methylguanine	Purines and purine derivatives	2.66 (1.33, 5.31)	5.5e-3	0.047

^1^ Metabolite sub-class information was obtained from the Human Metabolome Database.

^2^ Odds ratios (OR) and corresponding 95% confidence intervals (CI) are reported for a 1 SD increase in log-transformed metabolite levels, adjusted for sub-study endpoint, age, fasting status at blood draw, body mass index, race/ethnicity, diabetes mellitus, hypertension, menopausal status, menopausal hormone therapy use, smoking, dietary intake (DASH dietary adherence score), alcohol intake, physical activity, NSAID use, acetaminophen use, and persistent tinnitus.

^3^ *PE* Phosphatidylethanolamine.

^4^ *PC* Phosphatidylcholine.

We observed positive associations for 4 amino acid derivatives, including N6, N6-dimethyllysine (OR = 1.27, 95% CI: 1.07, 1.51), phenylacetylglutamine (OR = 1.27, 95% CI: 1.08, 1.48), homoarginine (OR = 1.82, 95% CI: 1.22, 2.73) and gabapentin (OR = 1.34, 95% CI: 1.11, 1.62); phosphatidylcholine PC(36:4)_B (OR = 1.75, 95% CI: 1.27, 2.42), PC (P-38:3)/PC (O-38:4) (OR = 1.47, 95% CI: 1.12, 1.94)), ribothymidine (OR = 1.98, 95% CI: 1.33, 2.95), 1-methylhistamine (OR = 1.62, 95% CI: 1.16, 2.25), 1-methylguanine (OR = 2.66, 95% CI: 1.33, 5.31). An inverse association was observed for phosphatidylethanolamine PE(P-38:5)/PE(O-38:6) (OR = 0.75, 95% CI: 0.62, 0.91).

The 10 metabolites had coefficients of variation (CV) that ranged from 9.5% to 54.3%. See Table S2 in the Supplement for quality metrics corresponding to the 10 metabolites associated with moderate or severe hearing loss. The results for all 278 metabolites from Models 1 and 2 can be found in Tables S3 and S4 in the Supplement.

**Fig. 1:**
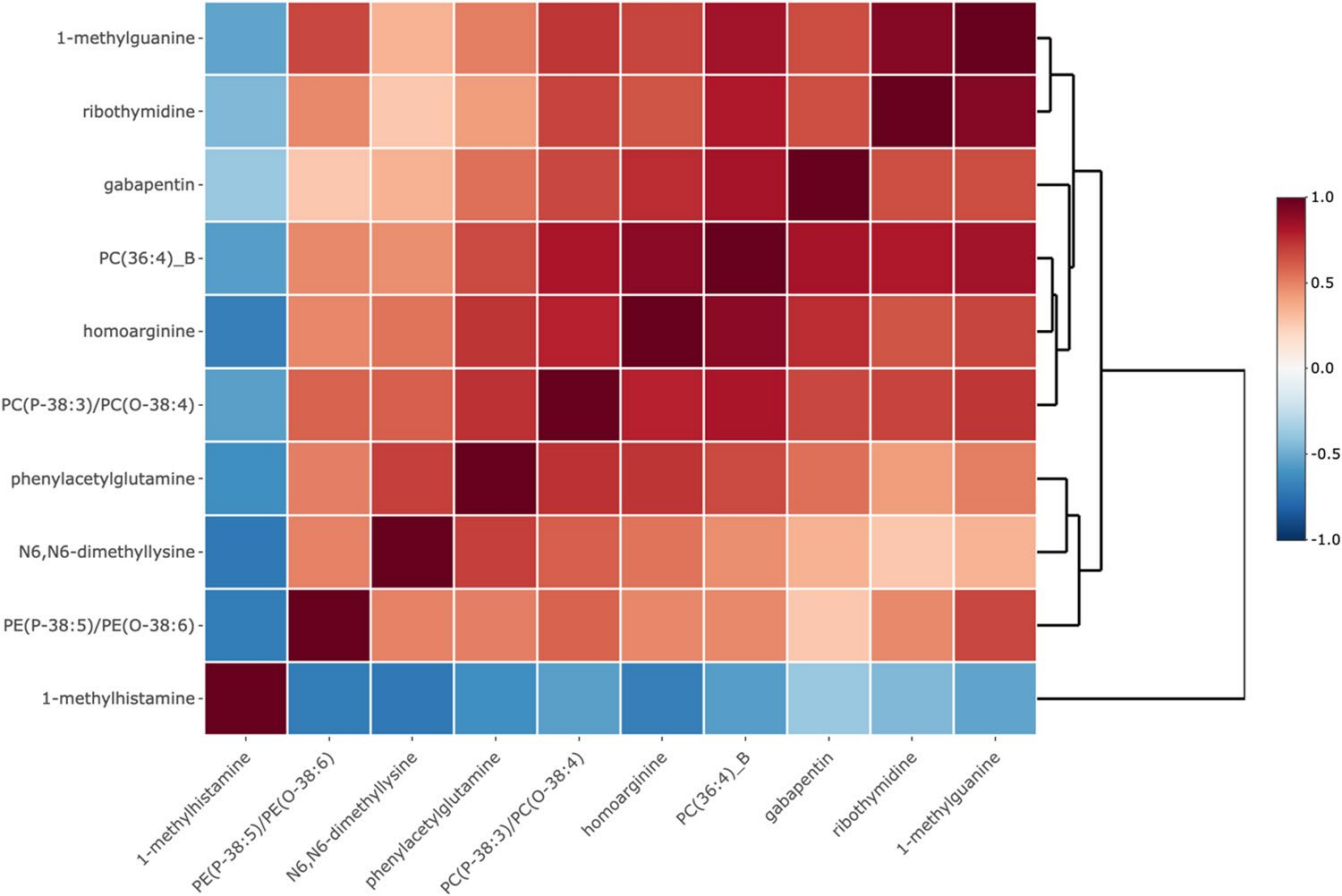
Pearson correlation network of 10 significant plasma metabolites. Red represents positive correlation, and blue represents negative correlation. *PE* Phosphatidylethanolamine, *PC* Phosphatidylcholine

The Pearson correlation network of the 10 significant metabolites is shown in Fig. 1. 1-methylhistamine was inversely correlated with each of the remaining 9 metabolites. All other metabolites were positively correlated with each other. The metabolite score included 9 of the 10 metabolites with weights ranging from − 0.57 for homoarginine to 0.47 for PC(36:4)_B (Table 3). Positive coefficients in the metabolite score correspond to metabolites in which high levels are associated with a higher odds of moderate or severe hearing loss and negative coefficients correspond to metabolites in which high levels are associated with a lower odds of moderate or severe hearing loss. In the fully adjusted model (Model 2), the metabolite score comprised of 9 metabolites was significantly associated with moderate to severe hearing loss; compared with women with no hearing loss, the OR for moderate or severe hearing loss was 1.56 (95% CI: 1.28, 1.91) per standard deviation increase in the metabolite score.

**Table 3:** Components of the metabolite Score: weights corresponding to individual plasma metabolites associated with hearing loss estimated in a Lasso logistic regression model

Metabolite	Metabolite Sub-class^1^	Coefficient	OR per 1 SD increase in metabolite levels^2,3^
PE^4^ (P-38:5)/PE(O-38:6)	Glycerophosphoethanolamines	−0.08	0.92
N6, N6-dimethyllysine	Amino acids, peptides, and analogues	0.34	1.40
phenylacetylglutamine	Amino acids, peptides, and analogues	0.42	1.52
gabapentin	Amino acids, peptides, and analogues	0.10	1.11
PC^4^(P-38:3)/PC(O-38:4)	Glycerophosphocholines	0.01	1.01
1-methylhistamine	Amines	0	1
PC^5^ (36:4)_B	Glycerophosphocholines	0.47	1.60
homoarginine	Amino acids, peptides, and analogues	−0.57	0.57
ribothymidine	Pyrimidine nucleosides	−0.21	0.81
1-methylguanine	Purines and purine derivatives	−0.02	0.98

^1^ Metabolite sub-class information was obtained from the Human Metabolome Database.

^2^ Odds ratios (OR) are per 1 SD increase in log-transformed metabolite levels, adjusted for sub-study endpoint, age, fasting status at blood draw and body mass index.

^3^ OR is calculated as the exponential of the estimated coefficient corresponding to each metabolite in the Lasso logistic regression model.

^4^ *PE* Phosphatidylethanolamine.

^5^ *PC* Phosphatidylcholine.

**Fig. 2:**
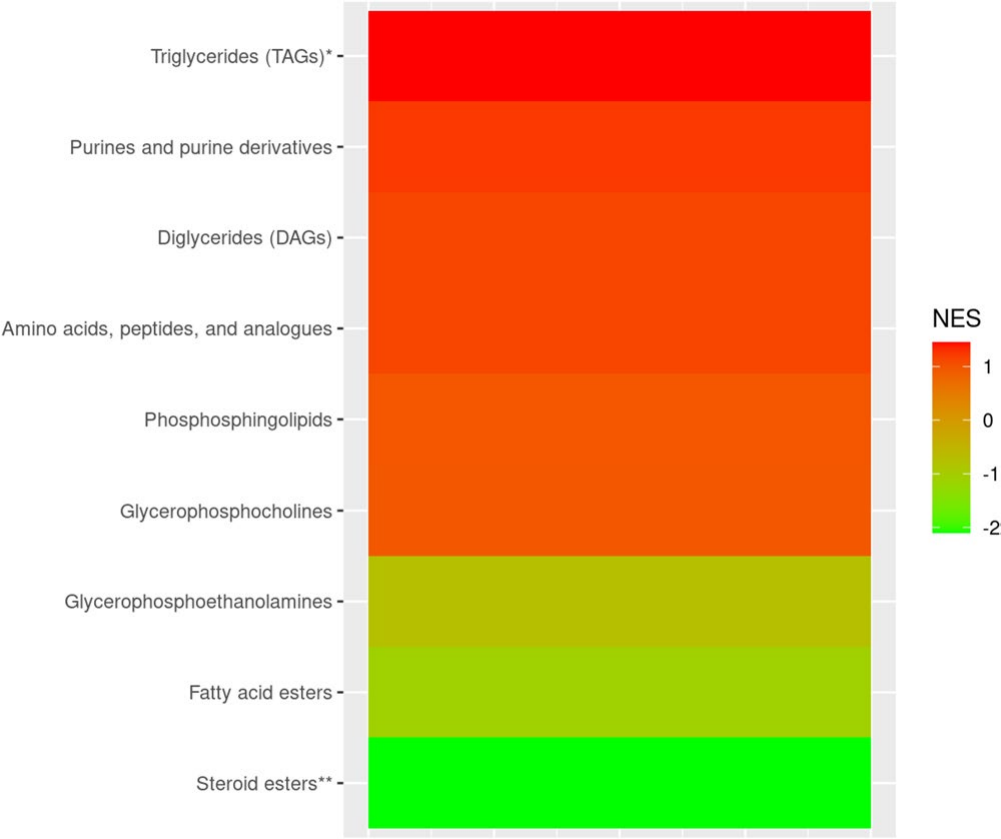
Metabolite Classes Associated with Moderate or Severe Hearing Loss in the Nurses’ Health Study. * indicates metabolite sub-classes with significant (q value < 0.05), positive normalized enrichment scores (NES). A positive NES indicates a set of metabolites that is enriched for positive associations, where higher metabolite levels are associated with moderate or severe hearing loss. ** indicates metabolite sub-classes with significant (q value < 0.05), negative normalized enrichment scores (NES). A negative NES indicates a metabolite set that is enriched for inverse (negative) associations, where lower metabolite levels are associated with moderate or severe hearing loss

**Fig. 3:**
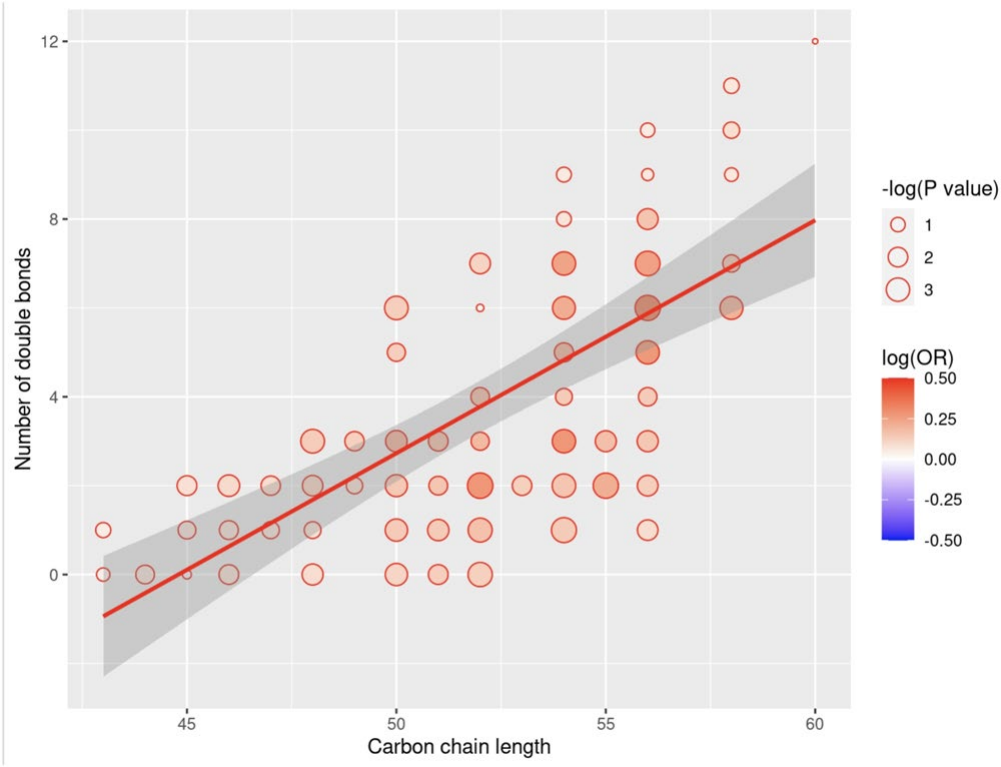
Carbon Chain Length and the Number of Double Bonds in Triglycerides and their Association with Moderate or SevereHearing Loss. Each datapoint represents one of 62 triglycerides, its size is proportional to the strength of its association with hearing loss measured by -log(P value), and is colored by the magnitude/direction of its association as measured by the logarithm of the odds ratio (log(OR)). The p value and OR are estimated in a logistic regression model with hearing loss as the outcome, the standardized levels of the triglyceride as the primary predictor, and adjusting for sub-study endpoint, age, fasting status at blood draw, body mass index, race/ethnicity, diabetes mellitus, hypertension, menopausal status, menopausal hormone therapy use, smoking, dietary intake (DASH dietary adherence score), alcohol intake, physical activity, NSAID use, acetaminophen use, and persistent tinnitus (Model 2)

In the MSEA, the set of 62 triglycerides (TAGs) were enriched for positive associations with hearing loss (q value < 0.05); and the set of 12 steroid esters were enriched for inverse associations with hearing loss (q value < 0.05) (Fig. 2). Among the 62 TAGs, carbon chain length was not associated with strength of significance of association with hearing loss (*p* = 0.38). However, TAGs with fewer double bonds were more enriched for statistically significant, positive asssociations with hearing loss when compared to TAGs with larger number of double bonds (*p* = 0.04) (Fig. 3).

In a sensitivity analysis conducted in a restricted sample that included participants in the LVS, the Racial Differences Study, and who served as controls in the case-control studies (i.e. did not develop the disease outcome in those studies), all 10 metabolites had significant independent associations with moderate or severe hearing loss (*p* < 0.05 in fully adjusted models) that were in consistent directions when compared with results from the primary analysis (Supplemental Table S5).

## Discussion

In this large population-based investigation of plasma metabolite profiles and hearing loss, we conducted a broad search for plasma biomarkers for self-reported moderate or severe hearing loss among women in a large well-characterized cohort using a high-throughput, agnostic metabolomics approach. Overall, 278 plasma biomarkers were assessed among 3925 participants. We identified significant associations for several individual metabolites and metabolite classes; 9 plasma metabolites were positively associated, and 1 metabolite was inversely associated with moderate or severe hearing loss. Triglycerides as a metabolite class were positively associated, while steroid esters were inversely associated with hearing loss. Triglycerides with fewer double bonds were more likely to be significantly associated with hearing loss when compared to those with larger number of double bonds. A lasso regression model was used to derive a metabolite score that was positively associated with odds of moderate or severe hearing loss.

Identifying plasma biomarkers for a multifactorial condition such as adult-onset hearing loss remains a substantial challenge. We identified significant associations with several novel metabolites and metabolite classes that suggest dysregulation of lipid metabolism, amino acid metabolism, and possibly other metabolic pathways, may influence auditory function. Our exploratory findings are hypothesis generating, and further investigations to replicate these findings and to uncover other potential associations are needed. Although differences in specific plasma markers of inflammation and lipid species among those with and without hearing loss have been shown, few studies have evaluated comprehensive plasma metabolomics profiles among individuals with hearing loss (Boullaud et al. 2022). Of the few previous metabolomics studies, most focused on changes in metabolite profiles following acoustic trauma or cisplatin-induced ototoxicity in animal models (He et al. 2017; Miao et al. 2022). For example, alterations in metabolites associated with oxidative stress were observed following acoustic trauma in rats (He et al. 2017)and in guinea pigs (Fujita et al. 2015). Human data on plasma metabolomics profiles and metabolic pathways and hearing loss are limited. A study in China of 62 individuals with noise-induced hearing loss and 62 controls observed significant differences in plasma metabolite profiles and metabolic pathways involved in glycerophospholipid, choline and fatty acid metabolism (Miao et al. 2021). A plasma lipidomics study among 185 adults with Alzheimer’s dementia found lower plasma phosphatidylcholine among those with self-reported hearing loss (Llano et al. 2020). Plasma metabolomics studies of other sensory, neurodegenerative and aging-related disorders have identified metabolic perturbations involving a range of metabolites and pathways, such as those involved in fatty acid, glycerophospholipid, sphingolipid, and amino acid metabolism, and suggest metabolic dysregulation may precede onset of clinically manifest disease (Bjornevik et al. 2019; Stoessel et al. 2018; Rojas et al. 2019).

In our study, higher plasma concentrations of two glycerophosphocholines, the phosphatidylcholine PC(36:4) and PC(P-38:3)/PC(O-38:4) were positively associated with hearing loss. PCs are essential components of cell membranes and lipoproteins and have an important role in membrane structure, cell signaling, energy metabolism and apoptosis. (Exton 1994; Cui et al. 1996; Cole et al. 2012)Higher plasma levels of certain PCs and lower plasma levels of others have been observed in several neurodegenerative disorders, including Parkinson’s disease, Alzheimer’s dementia and Huntington’s disease. (Stoessel et al. 2018; Cheng et al. 2016; Whiley et al. 2014).

We also observed that higher plasma phenylacetylglutamine was associated with hearing loss. Phenylacetylglutamine is a gut microbiota-derived metabolite, a product of bacterial phenylalanine metabolism (Moldave and Meister 1957), that may enhance platelet activation and thrombosis potential (Nemet et al. 2020). Higher plasma phenylacetylglutamine has previously been associated with adverse cardiovascular events, chronic kidney disease, diabetes mellitus and Parkinson’s disease (Stoessel et al. 2018; Cooper and Roncari 1989; Urpi-Sarda et al. 2019; Cirstea et al. 2020). Alterations in phenylalanine biosynthesis pathways were demonstrated in noise-exposed mice (Dong et al. 2013). Additional human studies of plasma phenylacetylglutamine and metabolites involved in phenylalanine metabolism are needed.

The significant association of gabapentin and hearing loss is an intriguing finding. There are several case reports of reversible or irreversible hearing loss with the use of antiepileptic drugs, including valproate, vigabatrin, carbamazepine and gabapentin (Hamed 2017). Gabapentin is a derivative of GABA and a γ-amino acid. It is prescribed as an anticonvulsant medication to treat focal seizures and for management of neuropathic pain. Off-label uses may include treatment of anxiety, bipolar disorder and specific sleep disorders. Gabapentin acts by binding to voltage-gated calcium channels and may inhibit inward calcium currents and attenuate neurotransmitter release (Hooft et al.,^2002^, and is a potent activator of voltage-gated potassium channels (Taylor 1997). Although gabapentin is a structural analog of GABA, it does not bind to GABA receptors and does not convert to GABA, bind to GABA receptors, or modulate GABA metabolism (Sills 2006). Gabapentin has been evaluated as a treatment for tinnitus, but evidence to support its effectiveness is lacking (Aazh et al. 2011). There is one published case report of reversible hearing loss and gabapentin use in the setting of acute renal failure (Pierce et al. 2008)and one case report of reversible hearing loss following an increase in pregabalin dose (Yilmaz et al. 2020). Further investigation of whether hearing loss may be associated with gabapentin or with its indications could be informative.

We observed that higher plasma homoarginine was associated with hearing loss. Homoarginine may increase nitric oxide, enhance endothelial function and may play a protective role in cardiovascular disease. Lower homoarginine was associated with higher risk of adverse cardiovascular outcomes and overall mortality, suggesting a cardioprotective role (Marz et al. 2010; Choe et al. 2013; Pilz et al. 2011); however, the influence of homoarginine on the central nervous system (CNS) is complex and not fully understood. Both high homoarginine and homoarginine deficiency may contribute to CNS disorders. (Choe et al. 2013; Pilz et al. 2011; Deignan et al. 2008, 2010; Chen et al. 2021) High homoarginine may be neurotoxic; severe neurologic and cognitive dysfunction were observed in animal studies and in humans with hyperargininemia, a rare autosomal recessive urea cycle disorder. Whether less extreme elevation of plasma homoarginine may influence neurodegenerative processes or auditory function is not known.

In contrast, we observed an inverse association between plasma levels of phosphaptidylethanolamine [PE(P-38:5)/PE(O-38:6)] and hearing loss. PEs are found in all living cells, composing 25% of all phospholipids, and are the most abundant phospholipid in the brain. Lower plasma PEs were observed among individuals with neurodegenerative conditions, such as Huntington’s disease (McGarry et al. 2021)and Alzheimer’s disease (AD) (Ginsberg et al. 1995)and in mouse models of early AD (Zhang et al. 2021). In the plasma lipidomics study among adults with AD, there was a suggestion that plasma PE was inversely associated with hearing loss among those with AD, but the association was not statistically significant (Llano et al. 2020).

In metabolite set enrichment analyses (MSEA) using pre-defined biologically meaningful sets of metabolites, we observed that plasma triglycerides (TAGs) were positively associated with hearing loss, while inverse associations was observed for plasma steroid esters. In addition, we observed that triglycerides with fewer double bonds were enriched for significant, positive associations with hearing loss; however, no such relationship was evident with carbon chain length. Elevated plasma triglycerides and chronic dyslipidemia have been associated with hearing loss in previous studies (Evans et al. 2006; Braffett et al. 2019; Simpson et al. 2013; Tan et al. 2018). Previous literature also suggests that triglycerides with fewer double bonds and shorter carbon chain lengths are associated with the risk of Type 2 diabetes and cardiovascular disease (Stegemann et al. 2014; Rhee et al. 2011). Evidence also suggests that alterations in plasma steroids, including androgens, estrogens, progestogens and corticosteroids, may influence auditory function (Curhan et al. 2017; Guimaraes et al. 2006; Kilicdag et al. 2004; Kim et al. 2002; Lee and Marcus 2001; Dubno et al. 2008), thus further investigation of specific steroidal metabolites and pathways, as well as lipid pathways, could be reveal potential targets for interventional studies.

In addition to identifying metabolite candidates for future investigations, this study illustrates methods that can be used effectively to identify a metabolomic fingerprint of adult-onset hearing loss and could be useful in studies of other auditory and aging-related disorders in which metabolic dysregulations contribute to their development and/or progression. To our knowledge, this was the first study to derive a composite metabolite score for hearing loss. To estimate a single metabolite score, or “metabolite fingerprint” of hearing loss, we conducted a lasso regression analysis based on the metabolites that were significantly associated with hearing loss in our multivariable-adjusted models. In this way, we were able to quantify the magnitude of the association of the set of selected metabolites with odds of moderate or severe hearing loss with a summary measure that considers the potential combined or synergistic contributions of multiple metabolites and metabolic pathways to the development of hearing loss.

Strengths of this study include the use of a richly characterized cohort that enabled adjustment for a broad range of covariates and a well-characterized metabolomics platform that measured a large set of metabolites with robust CVs and low missingness. Limitations include the assessment of only a subset of the full metabolome and the cross-sectional nature of metabolomic profiling that does not allow for the evaluation of temporal changes in metabolite profiles. Notably, previous studies of the stability of metabolite profiles in the NHS showed reasonable reproducibility over 1–2 years and over 10 years for 90% of the measured metabolites, with Spearman or intra-class correlation coefficients > 0.4 over 1–2 and over 10 years for most metabolites (Townsend et al. 2013; Zeleznik et al., 2022a, b). Information on hearing was obtained by self-report. Although pure-tone audiometry is the gold standard measure for evaluation of hearing loss, assessment of hearing loss based on self-report has been found to be reasonably reliable (Ferrite et al. 2011; Schow et al. 1990; Sindhusake et al. 2001). We chose a priori to examine moderate or severe hearing loss to minimize potential misclassification of the outcome. The sensitivity of a single question to detect moderate or severe hearing loss among women of similar age to our study population was shown to be high (95% and 100%, respectively). The analysis was conducted based on just one dataset, hence validation of the findings in independent datasets would be useful. Due to the lack of an independent replication dataset, the metabolite score estimate may have been subject to overfitting and thus cannot be interpreted as a validation of the metabolite associations. However, this estimate may be informative as a summary measure of the metabolite set association with hearing loss. Our study population included female health care professionals who were predominantly white, thus research in men and in additional populations of women is needed.

## Conclusion

In this large population-based investigation of plasma metabolite profiles and hearing loss, we identified several individual metabolites and metabolite classes that were significantly associated with self-reported moderate or severe hearing loss. We also derived a composite metabolite score for hearing loss, illustrating methods to identify a metabolite fingerprint of hearing loss that accounts for the potential combined effects of alterations in multiple metabolites and metabolic pathways. Additional studies to replicate these findings in independent datasets could provide important insights into the complex pathophysiologic processes underlying hearing loss and aging-related auditory dysfunction.

## Supplementary Information

Below is the link to the electronic supplementary material.

Supplementary Material 1

Supplementary Material 2

Supplementary Material 3

## Data Availability

The Nurses Health Study cohort welcomes new collaborations. Investigators interested in collaborations are invited to fill out a simple form that asks about the details of the collaboration https://docs.google.com/forms/d/e/1FAIpQLScAPV23ZIBpkk9CyEJ1OcFJjMol9elKEpLYnPu7g3PgBL57XA/viewform.
