# Supplementary material for: A plasma metabolomic fingerprint of moderate or severe hearing loss
Source: Metabolomics. 2026 Feb 27;22(2):31. doi: 10.1007/s11306-026-02395-8 (PMC12948882; doi:10.1007/s11306-026-02395-8)
Supplement: Supplementary file 3 — Supplementary Material 3 [file 11306_2026_2395_MOESM3_ESM.docx]

**Supplement to A Plasma Metabolomic Fingerprint of Moderate or Severe Hearing Loss**

**Statistical models**

Per metabolite logistic regression models

The association of each metabolite with moderate or severe hearing loss was assessed in logistic regression models with two sets of adjustment for confounders. The first model (Model 1) adjusted for age, fasting status, and BMI; a fully adjusted model (Model 2) further adjusted for race/ethnicity, diabetes mellitus, hypertension, menopausal status, menopausal hormone therapy use, smoking, dietary intake (DASH dietary adherence score), alcohol intake, physical activity, NSAID use, acetaminophen use, and persistent tinnitus. Since the analysis dataset was obtained was obtained by combining data from 14 sub-studies nested within NHS, all models were adjusted for a full set of sub-study endpoint indicators.

The analysis sample consisted of 3,925 women from the Nurses’ Health Study cohort who had available metabolomics data and reported their hearing status on the 2012 biennial questionnaire. The metabolomics data had been previously generated across 14 nested sub-studies and described in Table S1. Because these data came from multiple nested case–control studies, we accounted for the study design by including substudy-specific case–control indicators in the models. For each per-metabolite regression model, we adjusted for a categorical variable in which (1) participants grouped from the lifestyle validation and racial differences sub-studies served as the reference group; (2) included a study-specific category for each of two sub-studies (pancreatic cancer and ALS); and (3) included case vs. control categories corresponding to each of the remaining 10 sub-studies.

Multivariable-adjusted odds ratios (ORs) with 95% confidence intervals (95% CIs) were calculated for each metabolite. The false discovery rate was controlled at 5% using the q-value approach^1^. Metabolites that satisfied raw p and q value thresholds of 0.05 in Model 1 were then evaluated in Model 2. Statistically significant associations were identified for metabolites with p and q values < 0.05 in Model 2 (See Figure S1). The quality control metrics including coefficient of variation (CV) and intraclass correlation coefficient (ICC) for the metabolites meeting the threshold for statistical significance are shown in Table S2. The results for all 278 metabolites from Models 1 and 2 can be found in Tables S3 and S4 in the Supplement.

Lasso regression to derive a metabolite score

The Lasso regression analysis involved the following steps:

(1) Lasso regression: The weights associated with each metabolite in the metabolite score were derived in a Lasso regression model. This model included hearing loss as the binary outcome and the subset of significant metabolites as predictors, while adjusting for all sub-study indicators as described above. The penalty parameter was estimated using a 5-fold cross validation procedure^2^. The regression coefficients in the lasso regression were estimated in a dataset of 2873 participants, obtained after excluding participants with missing values in one or more of the significant metabolites. The lasso regression was fit using R packages *glmnet*.

(2) Calculation of a metabolite score: The metabolite score was derived as a weighted sum of the metabolites which were statistically significant in Model 2, where each metabolite weight corresponds to the coefficient that was estimated in the lasso regression model.

(3) Association of metabolite score with hearing loss: To obtain a summary measure of the metabolite set association with hearing loss, we evaluated the association between the metabolite score and moderate or severe hearing loss in a multivariate logistic regression model using the same dataset, adjusted for the same covariates and sub-study indicators that were included in Model 2. This model was fit to a dataset of 2666 participants, after excluding those with missing values in any of the metabolites in the metabolite score or in the covariates in Model 2.

Metabolite set enrichment analysis (MSEA)

We conducted a Metabolite Set Enrichment Analysis (MSEA)^3^ to identify specific metabolite classes that are enriched for concordant associations with moderate or severe hearing loss *^4^*. This method is used to identify and interpret patterns of metabolite concentrations in a biologically meaningful way. The metabolite set normalized enrichment score (NES) reflects the degree to which a set of metabolites within a metabolite class are overrepresented at the extremes (top or bottom) of the entire ranked list. Metabolites were ranked using the metric -log(p value)*log(OR)/SE, which combines both a measure of statistical significance as well as the magnitude and direction of the effect of hearing loss on metabolite levels. The statistical significance of the NES was obtained by the permutation test procedure with 1,000 iterations. The q-value approach was applied to control the false discovery rate at 0.05^1^. The metabolite sets were predefined by metabolite sub-classes according to the Human Metabolome Database (HMDB). Metabolite sets that contained fewer than 5 metabolites were excluded from the MSEA. We evaluated 9 sub-classes including triaglycerides (TAGs) (62 metabolites), steroid esters (12 metabolites), fatty acid esters (19 metabolites), diaglycerides (DAGs) (7 metabolites), purines and purine derivatives (5 metabolites), amino acids, peptides and analogues (44 metabolites), phosphosphingolipids (6 metabolites), glycerophosphoethanolamines (32 metabolites) and glycerophosphocholines (37 metabolites).

Relationship between carbon chain length. the number of double bonds, and hearing loss

We examined the relationship between carbon chain length. the number of double bonds, and hearing loss in the subset of 62 triglycerides. We fit linear models, using -log(p value) as the dependent variable, with carbon chain length and the number of double bonds as the predictors. The p value for each triglyceride was derived from Model 2, where hearing loss was the outcome and adjusted for the full set of confounders. Carbon chain length was categorized into 3 groups: 43-50, 51-54 and 55-60. Similarly, the number of double bonds was categorized into 3 groups: 0-2, 3-5 and 6-12. These grouping roughly ensured similar group sizes. We evaluated the statistical significance of carbon chain length and the number of double bonds using likelihood ratio tests.

Sensitivity analyses

To examine the possibility that the disease outcomes investigated in the case-control sub-studies from which these data were obtained may bias the metabolite associations with hearing loss, we conducted a sensitivity analysis that restricted the study sample to those participants in the Lifestyle Validation Study (LVS), the Racial differences study and those who served as the controls in the case-control sub-studies listed in Table S1. Results are shown in Supplemental Table S5.

**Supplementary Tables**

**Table S1** Sub-studies nested within the Nurses’ Health Study cohort.

| Sub-study | Number of Cases | Number of Controls | Not assigned^1^ | Number of Metabolites Measured |
| --- | --- | --- | --- | --- |
| Breast cancer | 490 | 459 | 0 | 306 |
| Ovarian cancer | 57 | 114 | 0 | 374 |
| Colon cancer  Pancreatic cancer | 135  1 | 148  65 | 0  0 | 309  86 |
| Diabetes | 338 | 356 | 0 | 330 |
| Stroke  Parkinson’s disease  Amyotrophic lateral sclerosis  Inflammatory bowel disease  Rheumatoid arthritis  Exfoliation glaucoma | 101  115  2  65  86  79 | 135  152  21  125  148  80 | 0  0  0  0  0  0 | 412  351  313  386  392  327 |
| Primary open-angle glaucoma | 163 | 172 | 0 | 316 |
| Lifestyle Validation Study^1^ | 0 | 0 | 235 | 359 |
| Racial differences study^1^ | 0 | 0 | 83 | 304 |

^1^ Sub-study was not designed as a case-control study.

**Table S2** Quality Control Metrics for 10 Plasma Metabolites Associated With Moderate or Severe Hearing Loss in the Nurses’ Health Study.

| Metabolite | Number of subjects in the per-metabolite analysis^1^ | Coefficient of variation (CV)^1^ | Intra-class correlation coefficient (ICC) ^2^ |
| --- | --- | --- | --- |
| PE(P-38:5)/ PE (O-38:6) | 3585 | 11.5% | 0.89 |
| N6,N6-dimethyllysine | 3585 | 16.6% | 0.99 |
| Phenylacetylglutamine | 3585 | 22.7% | 0.98 |
| Gabapentin | 3002 | 54.3% | 1.00 |
| PC^4^(P-38:3)/PC(O-38:4) | 3585 | 15.3% | 0.77 |
| 1-methylhistamine | 3314 | 40.6% | 0.99 |
| PC (36:4)_B | 3585 | 9.5% | 0.88 |
| Homoarginine | 3409 | 16.1% | 0.86 |
| Ribothymidine | 3585 | 25.9% | 0.36 |
| 1-methylguanine | 3273 | 27.5% | 1.00 |

^1^: Metabolite values missing for all participants in a sub-study were not imputed, as the missingness was due to the metabolite not being measured in the sub-study. In these instances, participants were excluded from the analysis of the specific metabolite.

**Table S3** Associations Between 278 Plasma Metabolites and Risk of Moderate or Severe Hearing Loss Among Women in the Nurses’ Health Study. Odds ratios (OR) and corresponding 95% confidence intervals (CI) are reported for a 1 SD increase in log-transformed metabolite levels, adjusted for sub-study endpoint, age, fasting status at blood draw and body mass index (Model 1).

File: TableS3_LogisticRegression-minimalmodel-partialimputation.xlsx

**Table S4** Associations Between 278 Plasma Metabolites and Risk of Moderate or Severe Hearing Loss Among Women in the Nurses’ Health Study. Odds ratios (OR) are per 1 SD increase in log-transformed metabolite levels, adjusted for sub-study endpoint, age, fasting status at blood draw, body mass index, race/ethnicity, diabetes mellitus, hypertension, menopausal status, menopausal hormone therapy use, smoking, dietary intake (DASH dietary adherence score), alcohol intake, physical activity, NSAID use, acetaminophen use, and persistent tinnitus (Model 2) .

File: TableS4_LogisticRegression-fullmodel-partialimputation-allmeta.xlsx

**Table S5** Individual Plasma Metabolites Associated with Moderate or Severe Hearing Loss in a Subset of 2,293 Nurses’ Health Study (NHS) Participants Sampled from the Lifestyle Validation Study (LVS), the Racial Differences Study, and controls from the other sub-studies listed in Table S1^1^.

| Metabolite | Metabolite Sub-class^2^ | OR^3^ (95% CI) | P-value |
| --- | --- | --- | --- |
| PE^4^(P-38:5)/PE(O-38:6) | Glycerophosphoethanolamines | 0.64 (0.49, 0.84) | 9.9e-4 |
| N6, N6-dimethyllysine | Amino acids, peptides, and analogues | 1.48 (1.17, 1.87) | 1.0e-3 |
| phenylacetylglutamine | Amino acids, peptides, and analogues | 1.34 (1.08, 1.65) | 7.9e-3 |
| gabapentin | Amino acids, peptides, and analogues | 1.38 (1.08, 1.76) | 1.0e-2 |
| PC (P-38:3)/PC (O-38:4)  1-methylhistamine | Glycerophosphocholines  Amines | 1.70 (1.20, 2.40)  1.57 (1.04, 2.35) | 2.8e-3  3.1e-2 |
| PC^5^(36:4)_B | Glycerophosphocholines | 1.68 (1.14, 2.47) | 9.0e-3 |
| homoarginine | Amino acids, peptides, and analogues | 2.46 (1.52, 3.99) | 2.6e-4 |
| ribothymidine | Pyrimidine nucleosides | 2.49 (1.55, 3.99) | 1.5e-4 |
| 1-methylguanine | Purines and purine derivatives | 2.74 (1.18, 6.37) | 1.9e-2 |

^1^ Participants who were cases in the sub-studies listed in Table S1 were excluded from the analysis.

^2^Metabolite sub-class information was obtained from the Human Metabolome Database

^3^Odds ratios (OR) and corresponding 95% confidence intervals (CI) are reported for a 1 SD increase in log-transformed metabolite levels, adjusted for sub-study endpoint, age, fasting status at blood draw, body mass index, race/ethnicity, diabetes mellitus, hypertension, menopausal status, menopausal hormone therapy use, smoking, dietary intake (DASH dietary adherence score), alcohol intake, physical activity, NSAID use, acetaminophen use, and persistent tinnitus

^4^ PE = phosphatidylethanolamine

^5^PC = phosphatidylcholine


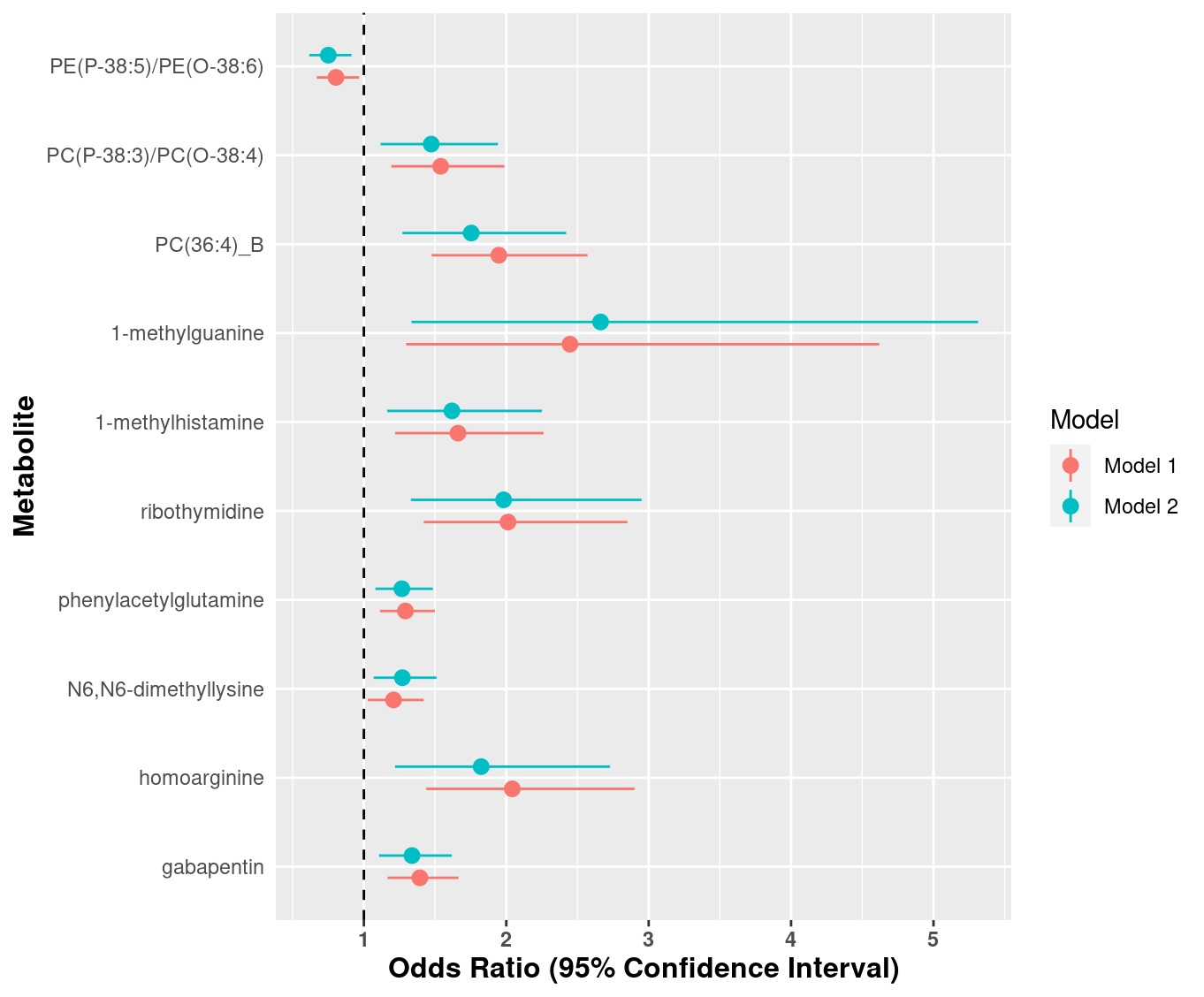


**Figure S1**: Individual Plasma Metabolites Associated with Moderate or Severe Hearing Loss Among Women in the Nurses’ Health Study.

Model 1 adjusts for sub-study endpoints, age, fasting status, and body mass index. Model 2 further adjusts for race/ethnicity, diabetes mellitus, hypertension, menopausal status, menopausal hormone therapy use, smoking, dietary intake (DASH dietary adherence score), alcohol intake, physical activity, NSAID use, acetaminophen use, and persistent tinnitus. PE = phosphatidylethanolamine. PC = phosphatidylcholine.

**References**

1. Storey JD, Tibshirani R. Statistical significance for genomewide studies. *Proc Natl Acad Sci U S A.* 2003;100(16):9440-9445.

2. Hastie T, Tibshirani, R., Friedman, J. *The Elements of Statistical Learning: Data Mining, Inference, and Prediction.* 2nd ed: Springer; 2009.

3. Subramanian A, Tamayo P, Mootha VK, et al. Gene set enrichment analysis: a knowledge-based approach for interpreting genome-wide expression profiles. *Proc Natl Acad Sci U S A.* 2005;102(43):15545-15550.

4. Gennady Korotkevich VS, Nikolay Budin, Boris Shpak, Maxim N. Artyomov, Alexey Sergushichev. Fast gene set enrichment analysis. *bioRxiv.* 2021.
